# Research on the Impact of Shot Selection on Neuromuscular Control Strategies During Basketball Shooting

**DOI:** 10.3390/s25134104

**Published:** 2025-06-30

**Authors:** Qizhao Zhou, Shiguang Wu, Jiashun Zhang, Zhengye Pan, Ziye Kang, Yunchao Ma

**Affiliations:** College of P.E. and Sports, Beijing Normal University, Beijing 100875, China; 17621061365@163.com (Q.Z.); 202211070024@mail.bnu.edu.cn (S.W.); 202422070046@mail.bnu.edu.cn (J.Z.); pan960806@163.com (Z.P.); 202421070023@mail.bnu.edu.cn (Z.K.)

**Keywords:** basketball shooting, muscle coordination, motor control, machine learning, shot selection

## Abstract

Objective: This study aims to investigate the effect of shot selection on the muscle coordination characteristics during basketball shooting. Methods: A three-dimensional motion capture system, force platform, and wireless surface electromyography (sEMG) were used to simultaneously collect shooting data from 14 elite basketball players. An inverse mapping model of sEMG signals and spinal α-motor neuron pool activity was developed based on the Debra muscle segment distribution theory. Non-negative matrix factorization (NMF) and K-means clustering were used to extract muscle coordination features. Results: (1) Significant differences in spinal segment activation timing and amplitude were observed between stationary and jump shots at different distances. In close-range stationary shots, the C5-S3 segments showed higher activation during the TP phase and lower activation during the RP phase. For mid-range shots, the C6-S3 segments exhibited greater activation during the TP phase. In long-range shots, the C7-S3 segments showed higher activation during the TP phase, whereas the L3-S3 segments showed lower activation during the RP phase (*p* < 0.01). (2) The spatiotemporal structure of muscle coordination modules differed significantly between stationary and jump shots. In terms of spatiotemporal structure, the second and third coordination groups showed stronger activation during the RP phase (*p* < 0.01). Significant differences in muscle activation levels were also observed between the coordination modules within each group in the spatial structure. Conclusion: Shot selection plays a significant role in shaping neuromuscular control strategies during basketball shooting. Targeted training should focus on addressing the athlete’s specific shooting weaknesses. For stationary shots, the emphasis should be on enhancing lower limb stability, while for jump shots, attention should be directed toward improving core stability and upper limb coordination.

## 1. Introduction

Shooting is the sole scoring method in basketball, and its effectiveness directly determines game outcomes [1]. The control of shooting involves a complex interplay between the central nervous system (CNS), which issues motor commands, and the peripheral system, which regulates execution force. Athletes must quickly transition from any initial posture to a shooting-ready stance within constrained time and space, synchronizing stable lower-limb extension with precise upper-limb force production [2]. This process demands tight coordination between the nervous and musculoskeletal systems [3], thereby elevating requirements for motor control. In a very short time, the CNS must overcome musculoskeletal redundancy and biomechanical constraints to select an optimal movement strategy. It then uses peripheral sensory feedback to compare actual and intended movements and promptly initiates error-correction mechanisms to ensure precision [4].

Table 1 summarizes recent analyses of athletes’ shooting mechanics [3,5,6,7,8]. Previous studies have identified key biomechanical features of shooting—namely, joint moments and the kinetic chain (i.e., dynamic relationships between joint moments and multi-joint movement sequences)—but suffer from two main limitations. First, they simplify neural control to individual muscle activity, neglecting the CNS’s modular control via muscle synergies. Second, they do not systematically compare neuromuscular drive patterns across shooting tactics (standing vs. jump shot). These conceptual gaps impair the development of neuroadaptive training interventions for specific shooting styles and impede a comprehensive understanding of distinct motor control strategies across tactics [9].

Motor commands are conveyed via multiple muscle synergies that coordinate specific muscle groups with defined weights and waveforms to execute movement tasks [10,11]. Movement arises from the neuromuscular system’s modular organization, which embeds musculoskeletal dynamics and task characteristics into a limited set of modules and uses their combination parameters to simplify motor control and learning [12,13,14]. The neural regulation of shooting also encompasses supraspinal signals, peripheral feedback, and the dynamic integration of central pattern generator (CPG) outputs, ultimately manifesting as α-motor neuron activation patterns across spinal segments [15]. Although this study involves elite basketball players, its primary goal is not to showcase their training superiority, but to support training optimization by analyzing how their neuromuscular control strategies differ across shooting modes. We aim to elucidate temporal and spatial variations in muscle synergy modules across different shooting choices (e.g., position and distance), thereby enabling coaches and athletes to tailor more effective training protocols for standing versus jump shots.

## 2. Materials and Methods

### 2.1. Research Subjects

Based on Pan Zhengye et al. (2024) [16], the reference sample size (f = 0.30, α = 0.05, power = 80%) was calculated using G*Power (v3.1.9.7; University of Düsseldorf, Düsseldorf, Germany). Following Matsunaga et al.’s (2018) [17] inclusion criteria, fourteen Level 1 basketball athletes with >7 years of training were recruited as the high-performance group (height: 192.07 ± 3.89 cm; mass: 87.14 ± 9.26 kg; age: 18.57 ± 1.28 years). All participants had to be free of neuromuscular or musculoskeletal injuries—and of any head or spinal cord trauma—within the preceding six months, and to use their dominant (right) hand for shooting. Before testing, the participants received a description of the study protocol and objectives and then provided written informed consent.

### 2.2. Experimental Instruments

#### 2.2.1. Three-Dimensional Infrared Motion Capture System

Kinematic data during shooting were captured using a Vicon 3D motion capture system (200 Hz; Vicon Motion Systems Ltd., Oxford, UK), comprising eight cameras (Vicon Motion Systems Ltd., Oxford, UK) and 39 reflective markers (14 mm diameter; Vicon Motion Systems Ltd., Oxford, UK) [18].

#### 2.2.2. Three-Dimensional Force Platform

Ground reaction force characteristics at initial ground contact during shooting were recorded using two embedded Kistler 3D force plates (1000 Hz; Kistler Instrumente AG, Winterthur, Switzerland; dimensions: 100.0 × 30.0 × 0.5 cm).

#### 2.2.3. Wireless Surface Electromyography (sEMG) Collection System

Surface EMG signals from the trunk and the dominant side’s 16 muscles were recorded using a 16-channel Delsys wireless EMG system (2000 Hz; Delsys, Inc., Natick, MA, USA). The monitored muscles were the flexor carpi radialis (FCR), biceps brachii (BB), triceps brachii (TB), anterior deltoid (AD), latissimus dorsi (LD), rectus abdominis (RA), erector spinae (ES), rectus femoris (RF), biceps femoris (BF), gluteus maximus (GM), tibialis anterior (TA), gastrocnemius lateralis (GL), gastrocnemius medialis (MG), soleus (SO), vastus medialis (VM), and vastus lateralis (VL).

### 2.3. Experimental Procedure

**Figure 1:**
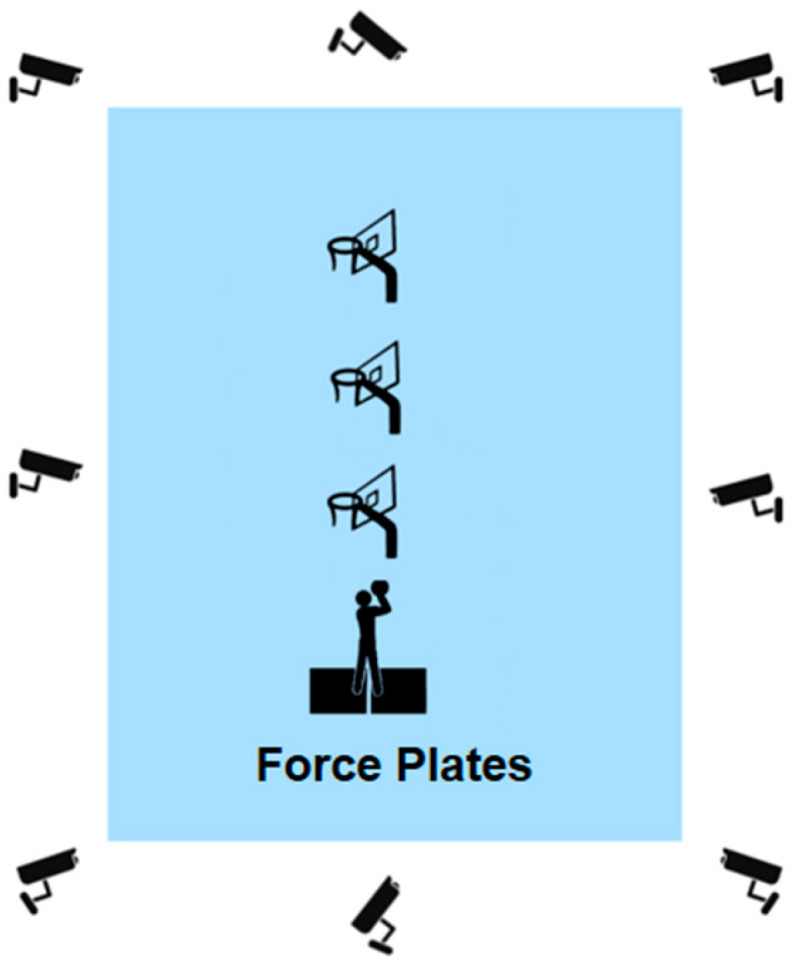
Simplified diagram of the laboratory layout.

Variations in movement conditions, such as jump height and shooting velocity, can disrupt established neuromuscular control strategies [15,16]. In the main experiment, the participants used their habitual shooting motions from training to perform both standing and jump shots, with shot type and distance randomized to ensure statistical validity. Considering typical competitive distances, three shooting ranges were selected: 2.45 m (near), 4.60 m (mid), and 6.75 m (far). During testing, the participants stood with one foot on each force plate and shot directly forward. After a thorough warm-up, formal testing commenced, and for each participant, kinematic and sEMG data were recorded for ten standing and ten jump shots (Figure 1).

### 2.4. Movement Phases

**Figure 2:**
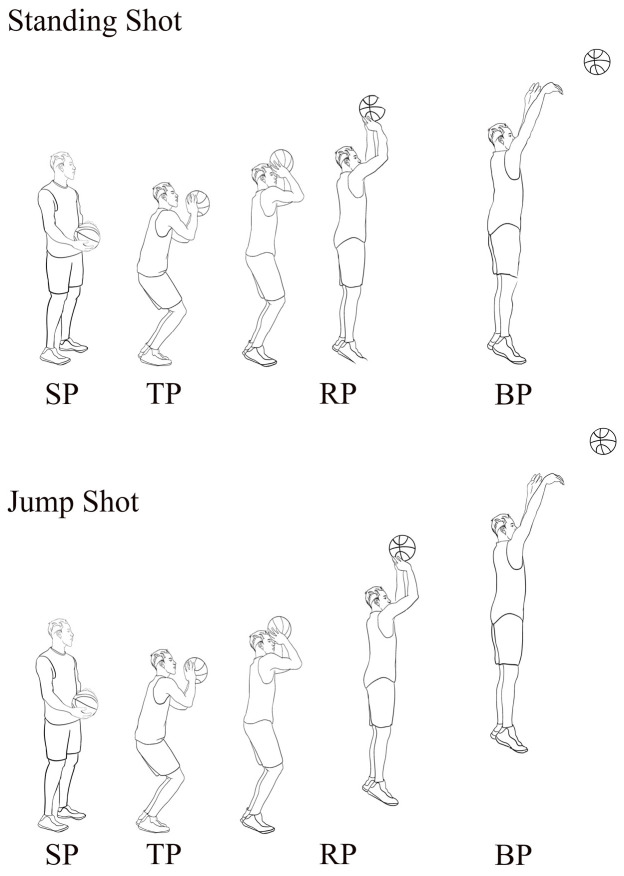
Different shooting techniques (original illustration by the authors).

The movement phases were defined as presented in Table 2 and Figure 2. The time courses of TP and RP were each normalized to 100% of their duration [16]. Furthermore, the instants when vertical ground reaction force (vGRF) exceeded 15 N and fell below 15 N were defined as ground-contact and take-off events, respectively, to calibrate the onset and offset of the TP phase [19].

### 2.5. Data Processing

This study employed the method of Pan Zhengye et al. (2023) [15] to systematically preprocess all biomechanical data. Kinematic parameters and ground reaction force (GRF) data were smoothed using a fourth-order low-pass Butterworth digital filter with a 20 Hz cutoff. Surface EMG signals (sEMG) were first high-pass-filtered at 50 Hz with a fourth-order zero-phase IIR Butterworth filter to remove baseline drift. The filtered signals were then full-wave-rectified to obtain absolute values. Next, a low-pass Butterworth filter with a 20 Hz cutoff was applied to extract the EMG signal’s linear envelope. Finally, EMG amplitudes were normalized to the [0, 1] range based on peak amplitudes recorded during each muscle’s maximum voluntary isometric contraction (MVIC) cycle.

#### 2.5.1. Spinal Segment Motor Output

The shooting action in basketball results from complex interactions among spinal-level processes, peripheral sensory feedback, and central pattern generator (CPG) signals. The resulting neural outputs can be characterized by the spatiotemporal activity patterns of α-motor neurons (MNs) [20]. Following the framework of Santuz et al. (2017) [21] and the myotomal mapping of Debra et al. (2006) [22], surface EMG signals from 16 muscles were mapped to the onset and offset locations of the α-MN pools in corresponding spinal segments, enabling the analysis of spinal motor output during shooting. Analyzing data at the level of spinal segments rather than individual muscles provides a more comprehensive view of MN pool activation. Although individual anatomical variations exist in myotomal distributions, these differences do not affect the validity of the mapping approach used in this study [20].

(1) $$S_{j}=\frac{\sum_{i=1}^{m_{j}}\left(\frac{k_{ji}}{n_{i}}\times {\mathrm{EMG}}_{i}\right)}{\sum_{i=1}^{m_{j}}\left(\frac{k_{ji}}{n_{i}}\right)}$$

In this equation, EMG*_i_* denotes the sEMG envelope of the *i*th muscle; *m_j_* is the number of muscles innervated by the *j*th spinal segment; *n_i_* is the number of spinal segments innervating the i th muscle; and *k_ij_* is the weighting coefficient between the *i*th muscle and its corresponding spinal segments, derived from the anatomical study by Scaleia et al. (2014) [23]. Equation (1) represents the extent of activation change for each spinal segment, from rest to peak activation. To account for differences in MN pool sizes across spinal segments, the values from Equation (1) are normalized by the number of motor neurons in each segment (MN*_j_*), thus adjusting the motor output of each MN pool [20].

#### 2.5.2. Muscle Synergy Extraction

This study applied classical Gaussian Nonnegative Matrix Factorization (NMF) to the preprocessed surface EMG (sEMG) signals to extract muscle synergy patterns. Following previous studies (Yang et al., 2020; Zhang et al., 2021) [24,25], the temporal structure of muscle synergies—termed Motor Primitives—refers to time-varying activation coefficients, while the spatial structure—termed Motor Modules—denotes the time-invariant relative weights of each muscle. The original muscle activation matrix *V* was constructed with 16 muscles (m = 16) as rows and 200 normalized time points (n = 200) as columns. The reconstructed matrix *V_r_* obtained from NMF decomposition can be expressed as follows [26,27]:

(2) $$V\approx V_{r}=MP$$

Here, *r* denotes the number of muscle synergies extracted by NMF; MMM is the motor module matrix (m × r); and PPP is the motor primitive matrix (r × n). The *M* matrix describes each muscle’s relative weight within the r synergies, while the *P* matrix captures their time-varying activation profiles. Together, the *M* and *P* matrices depict the muscle synergies underlying the shooting movement. The iterative estimation of *M* and *P* is performed via the expectation–maximization (EM) algorithm [19]:

(3) $$\left\{\begin{array}{l} P_{i+1}=P_{i}\frac{M_{i}^{T}V}{M_{i}^{T}VM_{i}P_{i}}\\ M_{i+1}=M_{i}\frac{V{\left(P_{i+1}\right)}^{T}}{M_{i}P_{i+1}{\left(P_{i+1}\right)}^{T}} \end{array}\right.$$

Convergence was defined as an R^2^ change of less than 0.01% between *V* and *V_r_* after 20 iterations [20]. To evaluate NMF reconstruction quality across synergy counts r (1–16), we used the Variance Accounted For (VAF). VAF indicates the proportion of variance in *V* explained by *V_r_* for *r* values from 1 to 16:

(4) $$\mathrm{VAF}=100\%\times (1-\frac{||V-V_{r}|{|}^{2}}{||V-\bar{V}|{|}^{2}})$$

We applied linear regression to model the relationship between VAF and *r*, selecting the elbow point—where the fitted curve’s slope changes most sharply—as the optimal synergy count. This synergy count maximizes the explained variance ratio of the original data with a minimal number of synergies [6].

We applied K-means clustering to muscle synergies derived from different shooting strategies to characterize their distinct features and to identify combined synergies. Combined synergies—formed by the fusion of two or more individual synergies—exhibit unclear functional roles and higher energetic costs; their relative abundance within the overall synergy repertoire reflects the extent of motor command modularity [18].

Furthermore, this study employed the Center of Activity (*CoA*) to characterize the activation distribution of muscle synergies across the three movement phases [20]. The *CoA* calculation is based on circular statistical methods and is described by the angular coordinate of the vector pointing to the circular centroid for each phase (where the polar coordinate direction represents phase and angle *θ* spans 0° to 360°):

(5) $$\left\{\begin{array}{l} A=\sum_{t=1}^{p}\left(\cos\theta_{t}\times P_{t}\right)\\ B=\sum_{t=1}^{p}\left(\sin\theta_{t}\times P_{t}\right)\\ CoA=\arctan(B/A) \end{array}\right.$$

Here, *p* represents normalized time points within each phase, and *P_t_* denotes the amplitude of the motor primitive at time *t*. To further characterize the duration of muscle synergies, we used the Full Width at Half Maximum (FWHM), defined as the total number of time points where the motor primitive’s amplitude exceeds half of its maximum. Together, CoA and FWHM describe the activation characteristics of synergies across both phases.

### 2.6. Statistical Analysis

All parameters are presented as mean ± standard deviation. Data normality was assessed using the Shapiro–Wilk test in SPSS (v27.0). Shooting accuracy and the proportion of combined synergies across shooting strategies were compared via chi-square and Fisher’s exact tests to determine the impact of shooting choice on accuracy and to explore whether movement patterns become increasingly modular with changes in shot distance or style. Additionally, one-dimensional statistical parametric mapping (SPM1d) in Python (v3.11.10) was used to perform repeated-measures ANOVA on spinal motor outputs, CoA, and FWHM across shooting strategies, providing an in-depth analysis of how neuromuscular control strategies vary with shooting choice.

## 3. Results

Table 3 shows the shooting accuracy of 14 high-level basketball players under different shooting choices. The chi-square test and Fisher’s exact test revealed no significant difference in shooting accuracy between the standing shot and jump shot at close range. However, as shooting distance increased, the shooting accuracy of the standing shot was significantly higher than that of the jump shot.

### 3.1. Spinal Segment Motor Output Characteristics

**Figure 3:**
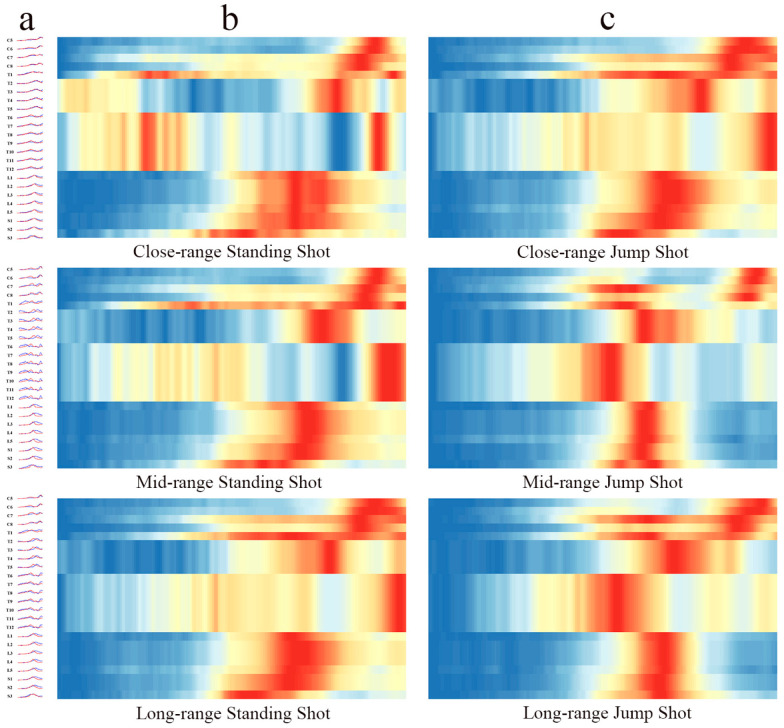
Spinal motor output features during the shooting phase. (**a**) Temporal characteristics of motor neuron pool (MN) output for each spinal segment (blue line: standing shot; red line: jump shot); (**b**) output amplitude of motor neuron pools (MNs) for each spinal segment during standing shots; (**c**) output amplitude of motor neuron pools (MNs) for each spinal segment during jump shots.

The spatiotemporal distribution characteristics of spinal motor output under different shooting choices are shown in Figure 3. A repeated-measures analysis of variance revealed significant differences in the C5-S3 segments under different shooting distances and techniques. These segments mainly control the upper limb, core, and lower limb muscle groups. Specifically, at close range, the activation level of the C5-S3 segment during the TP phase was significantly higher for standing shots than for jump shots, while during the RP phase, it was significantly lower for standing shots than for jump shots. At mid-range, the activation level of the C6-S3 segment during the TP phase was significantly higher for standing shots than for jump shots, while during the RP phase, it was significantly lower for standing shots. At long range, the activation amplitude of the C7-S3 segment during the TP phase was significantly higher for standing shots than for jump shots, whereas the activation level of the L3-S3 segment during the RP phase was significantly lower for standing shots than for jump shots (*p* < 0.01).

There were significant differences in the spatiotemporal distribution characteristics of spinal motor output under different shooting methods and distances, reflecting the distinct functions of the upper limb, core, and lower limb muscle groups. When comparing standing shots and jump shots, the main difference was not in the muscle activation level, but in the activation timing. During a standing shot, athletes activate their lower limb muscles early to maintain stability and continuous force generation. In contrast, during a jump shot, the focus is more on the explosive power of the kinetic chain, with force concentrated in the RP phase.

### 3.2. Muscle Synergy Characteristics

Table 4 shows the NMF reconstruction results and the proportion of combined synergies. As shooting distance increased, the proportion of combined synergies for both standing and jump shots significantly decreased (*p* < 0.05).

**Figure 4:**
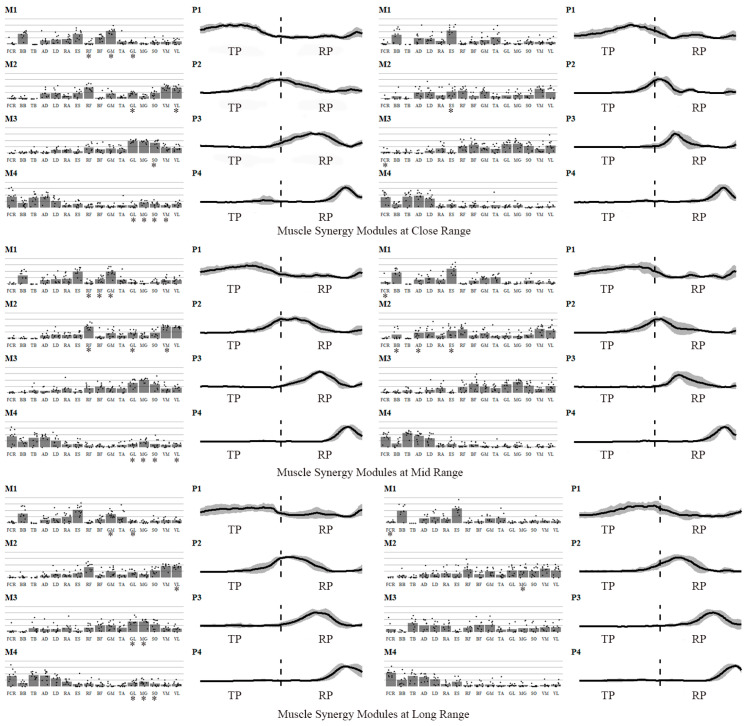
Muscle synergy modules for different shooting choices. Note: M1–M4 represent the four muscle synergy motor modules (spatial structure); P1–P4 represent the four muscle synergy motor primitives (temporal structure), with the vertical axis of the motor primitives standardized by the activation amplitude of each synergy. FCR: flexor carpi radialis; BB: biceps brachii; TB: triceps brachii; AD: anterior deltoid; LD: latissimus dorsi; RA: rectus abdominis; ES: erector spinae; RF: rectus femoris; BF: biceps femoris; GM: gluteus maximus; TA: tibialis anterior; GL: gastrocnemius lateralis; MG: gastrocnemius medialis; SO: soleus; VM: vastus medialis; VL: vastus lateralis. **Note:** * *p* < 0.05, Significantly higher than the contralateral side.

Figure 4 shows the temporal and spatial structures of muscle synergies for different shooting choices during the two phases. All six shooting choices involve four groups of muscle synergies. Group 1 synergy primarily occurs during the TP phase, with widespread activation of the biceps brachii, core, and lower limb muscles. This synergy helps maintain balance, control the basketball, and prepare for the descent of the center of mass. Group 2 synergy occurs during the transition from TP to RP, helping the body shift from a standing position to a squat and preparing for lower limb force production. A significant difference between standing and jump shots occurs at this stage. In standing shots, lower limb muscles primarily stabilize the body, while in jump shots, the erector spinae is activated first at close and mid-range distances, preparing the core muscles for rapid contraction to facilitate smooth take-off and force generation. Group 3 synergy occurs during the RP phase, primarily involving the lower limb muscles for push-off and force production. Group 4 synergy also occurs in the RP phase, involving the flexor carpi radialis and upper limb muscles, ensuring the direction and rotation of the basketball to improve shooting accuracy.

The spatiotemporal structures of synergy modules in standing shots differed significantly from those in jump shots at all distances. Specifically, Synergy 2 and Synergy 3 exhibited greater activation during the release phase (RP) in standing shots than in jump shots (*p* < 0.05). Furthermore, significant differences in muscle activation levels between standing and jump shots were observed across all shooting distances. At the near distance, standing shots showed significantly higher activation than jump shots for (1) Synergy 1—rectus femoris, gluteus maximus, and gastrocnemius lateralis (*p* < 0.01); (2) Synergy 2—gastrocnemius lateralis and vastus lateralis, with lower activation in erector spinae (*p* < 0.05); (3) Synergy 3—soleus (*p* < 0.01) and lower flexor carpi radialis activation (*p* < 0.05); and (4) Synergy 4—gastrocnemius lateralis, gastrocnemius medialis, soleus, and vastus lateralis (*p* < 0.01).

At mid-range, the muscle activation in Group 1 synergy (rectus femoris, gluteus maximus, biceps femoris) was significantly higher in standing shots than in jump shots, while flexor carpi radialis activation was significantly lower in standing shots (*p* < 0.05). For Group 2 synergy, rectus femoris, gastrocnemius lateralis, and vastus medialis activation was higher in standing shots, while biceps brachii, anterior deltoid, and erector spinae activation was significantly lower in standing shots compared to jump shots (*p* < 0.01). Gastrocnemius lateralis, gastrocnemius medialis, soleus, and vastus lateralis activation in Group 4 synergy was also significantly higher in standing shots than in jump shots (*p* < 0.05).

At long range, Group 1 synergy muscles (gluteus maximus and gastrocnemius lateralis) were more activated in standing shots, while flexor carpi radialis activation was significantly lower in standing shots compared to jump shots. For Group 2 synergy, vastus lateralis activation was significantly higher in standing shots than in jump shots, while gastrocnemius medialis activation was significantly lower in standing shots (*p* < 0.05). In Group 3 synergy, gastrocnemius lateralis and gastrocnemius medialis activation was significantly higher in standing shots than in jump shots (*p* < 0.05). For Group 4 synergy, gastrocnemius lateralis, gastrocnemius medialis, soleus, and vastus lateralis activation was also significantly higher in standing shots than in jump shots (*p* < 0.01).

Under various shooting methods and distances, the spatial structure of muscle synergy modules differed significantly, indicating that shooting methods markedly affect synergy module activation. During standing shots, lower limb muscle activation was higher, with athletes primarily relying on their lower limbs to maintain stability and generate push-off force. In contrast, during jump shots, core and upper limb muscle activation was higher, as athletes relied more on core strength and precise upper limb control.

**Figure 5:**
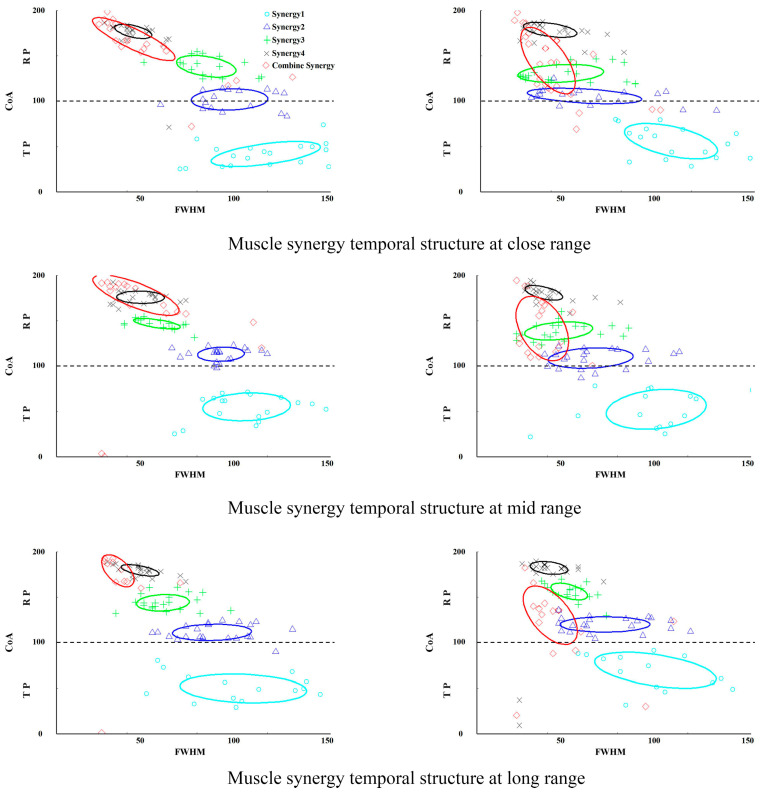
CoA-FWHM features of muscle synergy temporal structure during shooting (**left**: standing shot; **right**: jump shot).

The temporal activation profiles of muscle synergies during shooting are illustrated in Figure 5. While no significant temporal distribution differences were found between standing and jump shot synergy modules, standing shots exhibited lower combined synergy concentration than jump shots, indicating greater energy transmission efficiency. As shooting distance increased, the combined synergy proportion decreased significantly for both shot types, suggesting that long-distance shooting demands more efficient motor control and energy transmission. Athletes should therefore minimize reliance on combined synergies to clarify each synergy’s activation targets, reduce redundant movements, and optimize performance efficiency.

## 4. Discussion

This study derived muscle synergy modules and spinal motor outputs from sEMG signals to elucidate distinct neuromuscular control strategies across shooting modes and distances. The results indicated that, within a shooting range up to 6.75 m, standing shots achieved significantly higher accuracy than jump shots. Both shooting modes comprised four muscle synergy modules, whose spatiotemporal distributions aligned with the modular control theory (Yang et al., 2020) [24]. An analysis of spinal segment output amplitudes in conjunction with the spatial and temporal structures of muscle synergy modules revealed significant differences between shooting modes across distances:

(1) Differences in spinal output amplitudes across phases: Previous studies have focused on the number and spatial distribution of muscle synergies (e.g., d’Avella et al., 2003) [11], without linking these observations to spinal-level regulatory mechanisms. Our findings reveal distinct neural control demands for standing versus jump shots: standing shots require early postural stabilization, resulting in greater activation of spinal segments C5–S3 during the TP phase compared to jump shots, whereas jump shots emphasize explosive power, exhibiting significantly higher activation amplitudes in later segments (e.g., L3–S3; *p* < 0.01), thus demonstrating different force-generation patterns. This conclusion aligns with the spinal–muscle dynamic coupling model proposed by Pan et al. (2023) [18], which suggests that movement strategies optimize intermuscular coordination by modulating activation patterns encoded at the spinal level.
(2) Shot distance adaptability: As shooting distance increases, the proportion of combined synergies decreases significantly, consistent with the movement efficiency optimization hypothesis proposed by Santuz et al. (2017) [21]. In long-distance shooting, athletes enhance movement accuracy and energy utilization efficiency by reducing reliance on redundant synergy modules. This effect is particularly pronounced in jump shots and parallels findings by Zhang et al. (2021) in archery [25].
(3) Functional differentiation of synergy modules: Unlike Li et al. (2019) [2], who focused on upper-limb joint kinematics, this study uncovers significant functional differences in muscle synergy modules between standing and jump shots. For example, Synergy 1 during the TP phase exhibited widespread lower-limb activation, aligning with Konno et al. (2020) [6], both emphasizing lower-limb extension as the critical force-generation component in shooting. In contrast, Synergy 2 in standing shots (TP→RP transition) was dominated by lower-limb muscle activation, whereas jump shots at the same stage relied more heavily on core muscles (e.g., rectus abdominis and erector spinae). This divergence arises from distinct mechanical demands: standing shots depend on sustained lower-limb support for balance and stability, whereas jump shots require rapid core muscle contraction to facilitate smooth lift-off and force transmission. These findings also corroborate Bizzi et al.’s (2005) theory that muscle synergies adapt dynamically to varying task demands [13].

This study has several limitations: First, we did not compare neuromuscular control strategies between successful and missed shots; future research should address this gap. Second, this study examined only male athletes’ neuromuscular control strategies across shot types; subsequent studies should include female athletes to assess potential sex differences.

## 5. Conclusions

This study shows that changes in shooting technique and distance significantly affect neuromuscular control strategies during shooting. Targeted strategies should be adopted in training based on the athlete’s specific needs. For standing shots, emphasis should be placed on strengthening the rectus femoris, gluteus maximus, and gastrocnemius lateralis to enhance lower limb force generation and stability. For jump shots, the focus should be on developing explosive power in the upper limb and core muscles to improve upper limb accuracy and explosiveness during the shot. Additionally, specialized shooting training should be designed based on tactical roles, with specific plans for different shooting distances, aimed at selectively improving shooting precision.

## Figures and Tables

**Table 1:** Recent studies on basketball shooting.

Research Sources	Brief Research Content	Brief Research Findings
Fan et al. (2024) [3]	Differentiating changes in lower-limb synergy patterns between near- and far-distance shots using muscle synergies.	Shooting distance does not change the number of muscle synergies; however, it alters their activation timing and muscle involvement patterns.
Botsi et al. (2024) [5]	Comparing shot entry angle (EA), release time (RT), and shooting accuracy.	Jump shots exhibit larger entry angles (closer to the ideal 45°), whereas standing shots achieve higher accuracy.
Konno et al. (2024) [6]	Analyzing anticipatory postural adjustments.	Defensive perturbations disrupt anticipatory postural control mechanisms prior to jump shots, revealing the dynamic control system’s sensitivity in response.
Matsunaga et al. (2022) [7]	Analyzing differences in muscle synergy patterns between successful and unsuccessful shots.	Successful shots involve four muscle synergy modules, whereas missed shots involve only three.
Okubo et al. (2018) [8]	Analyzing the influence of vertical shoulder velocity and acceleration on ball release.	Jump shots require greater vertical fingertip acceleration to generate backspin; shoulder elevation can compensate for this additional demand.

**Table 2:** Definitions of movement phase delineation.

Movement Phase	Definition	Biomechanical Markers
Setup Phase, SP	Pre-shot ball-holding phase	Between ball acquisition and the onset of center-of-mass descent
Transformation Phase, TP	Descent of the body’s center of mass [6]	The initial decrease in shoulder marker height and the instant when vertical ground reaction force exceeded 15 N [6]
Rhythmical Phase, RP	The process of simultaneous lower-limb extension and upper-limb ball propulsion	The onset of the propulsive phase is defined as the moment when the flexion angle begins to increase. Furthermore, a vertical ground reaction force below 15 N marks the onset of the jump shot’s propulsive phase [6]
Ball Release Phase, BP	Release of the basketball from the athlete’s hands	The wrist joint attains its minimum flexion angle for the first time during the propulsive phase

**Table 3:** Shooting accuracy of the subjects.

Shooting Condition	Total Shots	Successful Shots	Shooting Accuracy	*p*
Close-range Standing Shot	140	117	83.6%	0.229
Close-range Jump Shot	140	108	77.1%	
Mid-range Standing Shot	140	99	70.7% *	0.020
Mid-range Jump Shot	140	93	66.4%	
Long-range Standing Shot	140	80	57.1% *	<0.001
Long-range Jump Shot	140	61	43.6%	

**Note:** * *p* < 0.05, and the same applies hereafter.

**Table 4:** NMF reconstruction results and combined synergy proportion.

	Close-Range Standing Shot	Close-Range Jump Shot	Mid-Range Standing Shot	Mid-Range Jump Shot	Long-Range Standing Shot	Long-Range Jump Shot
Minimum Number of Synergies	4.45 ± 0.58	4.82 ± 0.57	4.45 ± 0.58	4.59 ± 0.65	4.45 ± 0.50	4.41 ± 0.65
Reconstruction Quality (VAF)	0.94 ± 0.02	0.93 ± 0.02	0.94 ± 0.02	0.94 ± 0.02	0.95 ± 0.01	0.95 ± 0.02
Combined Synergy Proportion (%)	20.4%	25.4%	18.3% *	18.8% *	12.2% *	17.7% *

**Note:** * *p* < 0.05, Significantly lower than close-range shots.

## Data Availability

All data included in this study are available upon request by contact with the corresponding author.

## Abbreviations

The following abbreviations are used in this manuscript:

MDPI	Multidisciplinary Digital Publishing Institute
DOAJ	Directory of open access journals
TLA	Three-letter acronym
LD	Linear dichroism
